# Genetic Yield Gains In CIMMYT’s International Elite Spring Wheat Yield Trials By Modeling The Genotype × Environment Interaction

**DOI:** 10.2135/cropsci2016.06.0553

**Published:** 2017-01-12

**Authors:** Leonardo A. Crespo-Herrera, Jose Crossa, Julio Huerta-Espino, Enrique Autrique, Suchismita Mondal, Govindan Velu, Mateo Vargas, Hans J. Braun, Ravi P. Singh

**Affiliations:** 1 CIMMYT, Global Wheat Program, Apdo. 0660, Mexico City, México; 2 Institute Nacional de Investigaciones Forestales Agrícolas y Pecuarias, Campo Experimental Valle de Mexico, Apdo. Postal 10, 56230 Chapingo, Edo. de México, México; 3 Univ. Autonoma Chapingo, Carretera México-Texcoco Km. 38.5, Chapingo, 56230 Texcoco de Mora, México

## Abstract

We calculated the annual genetic gains for grain yield (GY) of wheat (*Triticum aestivum* L.) achieved over 8 yr of international Elite Spring Wheat Yield Trials (ESWYT), from 2006–2007 (27th ESWYT) to 2014–2015 (34th ESWYT). In total, 426 locations were classified within three main megaenvironments (MEs): ME1 (optimally irrigated environments), ME4 (drought-stressed environments), and ME5 (heat-stressed environments). By fitting a factor analytical structure for modeling the genotype **×** environment (G **×** E) interaction, we measured GY gains relative to the widely grown cultivar Attila (GYA) and to the local checks (GYLC). Genetic gains for GYA and GYLC across locations were 1.67 and 0.53% (90.1 and 28.7 kg ha^–1^ yr^–1^), respectively. In ME1, genetic gains were 1.63 and 0.72% (102.7 and 46.65 kg ha^–1^ yr^–1^) for GYA and GYLC, respectively. In ME4, genetic gains were 2.7 and 0.41% (88 and 15.45 kg ha^–1^ yr^–1^) for GYA and GYLC, respectively. In ME5, genetic gains were 0.31 and 1.0% (11.28 and 36.6 kg ha^–1^ yr^–1^) for GYA and GYLC, respectively. The high GYA in ME1 and ME4 can be partially attributed to yellow rust races that affect Attila. When G **×** E interactions were not modeled, genetic gains were lower. Analyses showed that CIMMYT’s location at Ciudad Obregon, Mexico, is highly correlated with locations in other countries in ME1. Lines that were top performers in more than one ME and more than one country were identified. CIMMYT’s breeding program continues to deliver improved and widely adapted germplasm for target environments.

Wheat is one of the most important food crops in the world, as it provides 20% of the total energy and protein in the human diet (Food and Agriculture Organization, 2016). The harvested wheat area in 2014 was 221.6 million ha, which accounted for about 30% of the global harvested cereal area, with a production volume of nearly 729 Tg of wheat grain (Food and Agriculture Organization, 2016). Mainly because of the changing climate and increasing food demands, wheat production faces several challenges, namely sustainably increasing global grain yields by 2 to 3% annually and protecting yield gains from insects and diseases (Hawkesford et al., 2013). Breeding wheat with high and stable yield potential and resistance or tolerance to biotic and abiotic stresses, along with consumers’ preferred processing quality, is of paramount importance in the current global scenario. In addition to the breeding efforts, it is also important to develop new cropping technologies and systems that facilitate the expression of the genetic potential of new wheat cultivars in farmers’ fields.

The Global Wheat Breeding Program at CIMMYT was founded to explore strategies for breeding widely adapted and highly stable wheat cultivars (Braun et al., 1996; Singh and Trethowan, 2007). These strategies include: (i) the exchange and selection of segregating populations in contrasting environments (shuttle breeding), (ii) multilocation testing at representative sites in the environments where wheat is grown, and (iii) germplasm evaluation under stressed and optimal conditions. Yield stability and wide adaptation are increasingly important, as the climate at specific locations becomes more variable over the years. Additionally, smallholder farmers often cannot afford to grow a set of agronomically diverse varieties for controlling risk, tending to grow single varieties that, over the years, have proven to be stable. Smallholder farmers need varieties that can cope with a highly variable climate at their locations, such as varieties with high yield potential to take advantage of rain and irrigation when available and also with heat and drought tolerance for dry, hot years, which are often stresses that extend to large geographical areas and thus are common to farmers from different countries.

As part of its multilocation testing strategy, CIMMYT distributes annual nurseries and replicated yield trials through an international collaborative network that includes more than 300 cooperators. Such nurseries consist of newly developed lines targeted at certain environments (e.g., the ESWYT for optimal environments) or at achieving certain breeding goals. Cooperators collect and send the data back to CIMMYT. This information allows breeders to make better crossing and selection decisions. One important key for the global wheat breeding program at CIMMYT is the concept of the ME, introduced in 1988 to describe target environments globally (Rajaram et al., 1993; Braun et al., 1996). Megaenvironments are defined as geographical regions that share similar abiotic and biotic constraints, production needs, consumer preferences, and productivity levels. They are not necessarily contiguous, can occur in several countries, and are frequently transcontinental (Rajaram et al., 1993). The initial characterization of MEs was done in a more qualitative manner; later, because of the development of geographic information systems, it was possible to define MEs in a more quantitative way (Hodson and White, 2007; Braun et al., 2010). Twelve MEs for wheat production have been defined, of which ME1 to ME6 correspond to spring wheat and ME7 to ME12 to facultative and winter wheat. Detailed descriptions of these MEs can be found in Rajaram et al. (1993), Hodson and White (2007), Braun et al. (2010), and in an internet-based platform launched by CIMMYT (www.wheatatlas.org, accessed 10 Oct. 2016).

Megaenvironment 1 comprises areas with optimal conditions: low rainfall but irrigated, with an average minimum temperature in the coolest quarter between 3 and 11°C. Megaenvironment 4 has low rainfall and the wettest quarter, with average precipitation between 100 and 400 mm; its major abiotic stress is drought. Megaenvironment 5 is characterized by its tropical high rainfall but can also be irrigated; the major abiotic constraint is heat stress, with an average minimum temperature of 11 to 16°C during the coolest quarter. It is estimated that in 2014, about 47.2 million ha of wheat were grown in ME1, 13.5 million ha in ME4, and 2.1 million ha in ME5 (Lantican et al., 2016). Wheat is autumn-sown in these three MEs. Testing locations can also be assigned to a specific ME based on agronomic practices such as planting date and irrigation practices. This information is relevant because it is possible to simulate different MEs by modifying sowing dates and water management in certain locations. For instance, at CIMMYT’s experimental station in Ciudad Obregon, Mexico, it is possible to simulate ME5 by sowing late so the plants will be heat stressed and to simulate ME4 by reducing the amount of water or irrigation events so the plants will grow under drought stress conditions.

Sharma et al. (2012) quantified the genetic yield gains for the ESWYT that were targeted mainly at sites in ME1 distributed over 919 locations in 69 countries during 1995 to 2010. Genetic gains were determined by regressing the differences in mean yields of the five highest yielding entries on the mean trial yield and the mean yield of the cultivar Attila. Overall, the mean yield of the five highest yielding entries showed an annual gain of 0.65% over that of Attila, whereas the yearly gains for ME1, Egypt, India, and Pakistan were 0.55%, 1.13, 0.83, and 0.5%, respectively.

Differential phenotypic response across a series of environments generate a G × E interaction, which can take the form of a crossover or noncrossover interaction. It is possible to model the G × E interaction by directly fitting a structure that uses the leading principal components of a covariance matrix, as in the factor analytic (FA) model (Crossa et al., 2006; Burgueño et al., 2008; Meyer, 2009). The FA model is an efficient and flexible procedure for reducing the dimensionality and complexity of the variance–covariance matrix that combines the main effects of genotypes and the G × E interaction (Crossa and Cornelius, 1997). Crossa et al. (2006) showed that by fitting a FA structure, it is possible to model the effects of genotypes and the G × E interaction efficiently; by doing this, the precision of the prediction of breeding values increases.

In this study, 8 yr of historical data derived from international ESWYTs were analyzed by fitting the FA model structure in each of the ESWYTs established during those years in three MEs. Our objectives were: (i) to estimate the yield gains for ESWYT germplasm in ME1, ME4, and ME5; and (ii) to identify the most recent high-yielding lines developed by CIMMYT’s Global Wheat Program for ME1 in the 34th ESWYT.

## MATERIALS AND METHODS

### Data

The ESWYT is a nursery annually distributed by CIMMYT to international collaborators, predominantly in developing countries. The main target of ESWYT-selected germplasm is ME1. However, because of CIMMYT’s international collaborative network, ESWYTs are distributed to regions classified within other MEs (for instance, ME4 and ME5) because cooperators in those MEs also find locally adapted lines from the ESWYTs.

The ESWYTs consist of 50 lines selected after 2 yr of testing under optimal irrigation conditions. The lines are tested for an additional 1 to 2 yr under drought and heat stressed conditions at CIMMYT’s experiment station in Ciudad Obregon, Mexico. The first slot of the entries is assigned to a local check (LC), which is often the best commercial variety or a new varietal candidate at each location where the ESWYTs are distributed. The LC entry is selected by the cooperators. Four CIMMYT checks are included in each location and they can change from year to year. However, from 2006 to 2014, spring wheat cultivar Attila (released as PBW343 in India, where it is widely grown) has been consistently included in the trials. The rest of the lines are new CIMMYT-derived cultivars released in different countries. The trial design is an α-lattice with two replicates. Cooperators follow the local management practices. From all the data returned and considered for the analysis, 5% of the trials were treated with fungicides and 74% were fertilized; in 45% of the trials, weeds were controlled with herbicides. For additional information on agronomic practices and the main characteristics of the local checks used for each site we encourage the reader to visit the webpage of the International Wheat Improvement Network (http://www.cimmyt.org/international-wheat-improvement-network-iwin/, accessed 10 Oct. 2016), where the information is freely available or is free on request.

We analyzed the yield data (in Mg·ha^–1^) for the 8 yr from 2006–2007 (27th ESWYT) to 2014–2015 (34th ESWYT). Throughout this period, yield and agronomic data for the trials were reported at 426 locations all over the world, which is about a 50% recovery rate for the results. The locations in each year were assigned to a specific ME according to their average historical climate data (minimum temperatures and precipitation). The trial management information provided by cooperators (if available) was also used to assign MEs (i.e., irrigated vs. nonirrigated, number of irrigation events, amount of water provided to the trial, and sowing date). The ME assignment was then refined by performing a cluster analysis of each ESWYT to distinguish between high- and low-yielding environments. The locations used for the statistical analysis were those included in ME1, ME4, and ME5.

### Statistical Analysis

All statistical analyses were performed with ASREML-R (Butler et al., 2009) in R version 2.15.3 software (R Development Core Team, 2013). Data from each ESWYT were analyzed in four ways: (i) an analysis by location, (ii) a combined analysis across locations grouped by ME, (iii) a combined analysis across all locations and all MEs, and (iv) a combined analysis across ME1 locations in certain countries (India, Pakistan, and Egypt) but only for the 34th ESWYT.

### Mixed Models

#### Single-environment model

The linear mixed model used for the individual location analysis of the *i*th genotype, in the *r*th replicate within the *k*th sub-block (*Y_ijk_*) is:

(1) $$Y_{ijk}=\mu +R_{j}+SB_{k}\left(R_{j}\right)+G_{i}+\epsilon_{ijk}$$

where μ is the general mean; *G_i_* is the fixed effects of the genotypes (*i* = 1,…,50); *R_j_* is the fixed effects of the replicates (*j* = 1, 2); *SB_k_* denotes the random effects of the sub-blocks (*k* = 1,…,5), which are assumed to be independently and identically normal distributed with a mean of zero and a variance of σ*^2^_sb(r)_*; and ε*_ijk_* is a random residual assumed to be independently and identically normal distributed with a mean of zero and a variance of σ*^2^*_ε_. Heritability was estimated from the variance components of the individual site analyses to discard all locations with a heritability lower than 0.05 from the combined analyses.

#### Linear Mixed Model for Multienvironment Trial Analysis

The mixed model used for fitting the data from *g* genotypes, *s* sites and *r* replicates in each site is shown in Eq. [1]:

(2) $$Y=Xb+Z_{r}r+Z_{g}g+Z_{ge}ge+e$$

where **X** is the incidence matrix for the fixed effects of sites and **Z_r_, Z_g_**, and **Z_ge_** are the incidence matrices of zeros and ones for the random effects of replicates within sites, genotypes, and G × E interactions, respectively. The vector **b** denotes the fixed effect of sites; the vectors **r**, **g**, **ge**, and **e** contain the random effects of replicates within sites, genotypes, G × E interactions, and residuals, respectively, and are assumed to be random and multivariate normally distributed with a zero mean vectors and the variance–covariance matrices **R**, **G**, **GE**, and **E,** respectively, that is,

$$\left[\begin{array}{c} r\\ g\\ ge\\ e \end{array}]∼\text{N}([\begin{array}{c} 0\\ 0\\ 0\\ 0 \end{array}]+[\begin{array}{cccc} R & 0 & 0 & 0\\ 0 & G & 0 & 0\\ 0 & 0 & GE & 0\\ 0 & 0 & 0 & E \end{array}]\right)$$

where the variance of the response vector **Y** is:

$$V\left(Y\right)=Z_{r}{RZ}_{r}^{\prime }+Z_{g}{GZ}_{g}^{\prime }+Z_{ge}{GEZ}_{ge}^{\prime }+E.$$

The variance-covariance matrices **R, G,** and **E** are assumed to have a simple variance–covariance component structure such that **R** = ∑_r_ ⊗ **I_r_**, where ∑_r_ = diag (σ^2^_r_j__, j = 1,2,…..*s*); **I**_r_ is the identity matrix of order *r*, **E**=σ^2^_e_ **I** (where σ^2^ is the independent and identical error variance); and **G**= σ^2^_g_**I**, where σ^2^_g_ is the genetic variance, assuming that there is no relationship between lines.

The **GE** = ∑_ge_ ⊗ **I** interaction matrix variance–covariance can be represented as:

(4) $$GE=\Sigma_{ge}⊗I=[\begin{array}{cccc} {\sigma^{2}}_{{ge}_{1}} & \rho_{12}{\sigma ge}_{1}{\sigma ge}_{2} & \ldots & \rho_{1s}{\sigma ge}_{1}{\sigma ge}_{s}\\ \rho_{12}{\sigma ge}_{1}{\sigma ge}_{1} & {\sigma^{2}}_{{ge}_{2}} & \ldots & .\\ . & . & \ldots & .\\ . & . & \ldots & .\\ . & . & \ldots & .\\ \rho_{s1}{\sigma ge}_{s}{\sigma ge}_{1} & . & \ldots & {\sigma^{2}}_{{ge}_{s}} \end{array}]⊗I$$

where the *j*^th^ diagonal element of the *s* × *s* matrix σ^2^ *ge*_j_is the G × E variance σ^2^*_ge j_* in the *j*^th^ site. The *ij*^th^ element is the G × E variance covariance ρ_ij_σ*_ge_*_i_ σ_ge j_ between Sites *i* and *j*; thus ρ_ij_ is the correlation of G × E variance effects between Sites *i* and *j*. The matrix **I** is of order *g* × *g* and assumes that the lines are not related and the breeding value of each genotype will be predicted only by the value of the empirical response of the genotype itself.

Different structures can be used to model the matrix **GE**. The most restrictive variance–covariance structure is to assume that **GE** variances within all sites are equal and that all pairwise correlations between genotypes and between sites are zero. On the other hand, the most liberal structure is the completely unstructured model which assumes matrix **GE** contains *s*(*s*-1)÷ 2 parameters. In this study, we used the FA structure that models covariance among observations in terms of a few hypothetical unobserved factors, which is useful for modeling the matrix **GE**. The FA model has been extensively used for modeling **GE** (Smith et al., 2002; Crossa et al., 2004) and a full description of FA can easily be found in several publications.

Best linear unbiased predictors (BLUP) were obtained for each genotype in the analysis. We expressed the BLUP of the GY of the 10% highest-yielding lines (HYL) of each ESWYT in terms of (i) the spring wheat cultivar Attila (GYA) and in terms of (ii) the LCs. These proportions were regressed on the years to estimate the annual yield gains in relation to Attila and the LC. We also performed multienvironment trial analyses by computing the BLUPs without modeling the G × E interactionand also performed the regression of the best 10% highest yielding lines in each ESWYT.

#### Analyses of the 34th ESWYT

Since the 34th ESWYT was the trial most recently distributed by CIMMYT at the time of this study, it was further analyzed to identify the most recent wheat lines with higher yield potential in the target environment. Site regression analysis, as described by Crossa and Cornelius (1997) and Crossa et al. (2002), was performed on the scaled data of the country means with locations assigned to ME1 to identify G × E patterns and correlations between countries. Biplots were made with the R package GGEBiplotGUI in R version 3.2.4. Genotypes are represented by numbers in the biplots; their corresponding matches can be found in the Supplementary Material.

## RESULTS

**Fig. 1:**
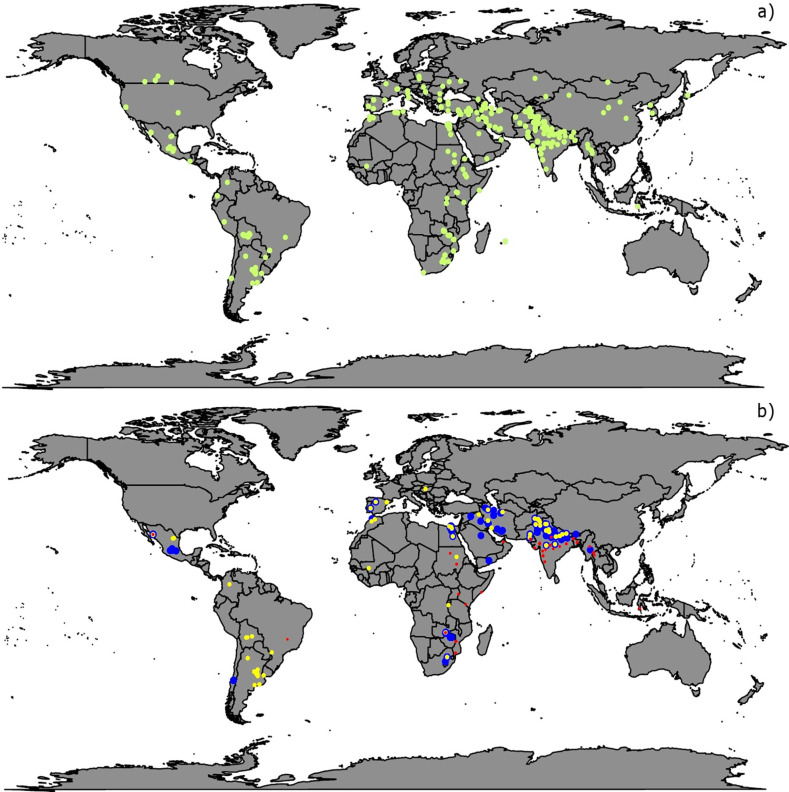
(a) Locations where Elite Spring Wheat Yield Trials (ESWYT) data were returned from 2006–2007 (27th ESWYT) to 2014–2015 (34th ESWYT); (b) locations assigned to Megaenvironment 1 (ME1) (blue), ME4 (yellow), and ME5 (red).

Based on all the data returned from 426 locations throughout the world, 305 locations were analyzed and classified as ME1, ME4, and ME5 on the basis of their climate data and agronomic practices, representing a wide range of environments (Table 1; Table 2; Fig. 1). The remaining 121 trials were not analyzed because they had low heritability (<0.05) or because they were not classified in any of the considered MEs. Locations within ME2 were not analyzed because there were usually less than three on a yearly basis. Generally, GYs were higher in ME1, followed by ME5 and ME4.

**Table 1:** Percentage of sites in geographical regions providing data for 27th to 34th Elite Spring Wheat Yield Trials.

Geographical region	Percentage
Eastern and Southeastern Africa	12.9
North Africa	7.1
West Asia	19.0
South Asia	21.8
East Asia	3.4
North America	9.9
South and Central America	12.9
Eastern Europe	7.5
Western Europe	5.4

**Table 2:** Number of locations per Megaenvironment (ME) in which the 27th to 34th Elite Spring Wheat Yield Trial (ESWYTs) were evaluated and analyzed.

ESWYT									
	27th	28th	29th	30th	31th	32th	33th	34th	Total
ME1	26	14	19	21	25	24	26	25	180
ME4	4	9	9	8	12	7	9	12	70
ME5	6	5	5	7	7	7	12	6	55
Total	36	28	33	36	44	38	47	43	305

**Fig. 2:**
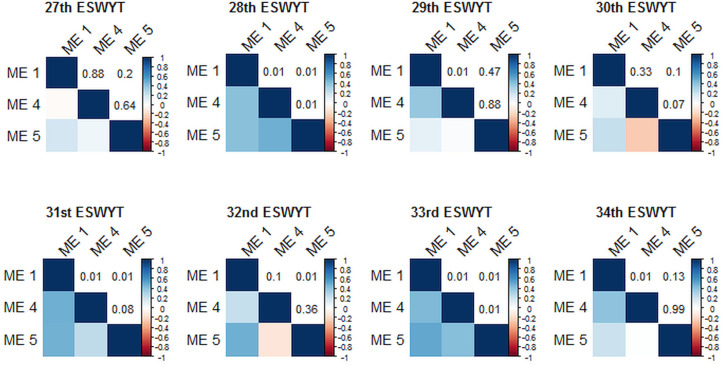
Phenotypic correlations (below the diagonals) between Megaenvironment 1 (ME1), ME4, and ME5 in each evaluated Elite Spring Wheat Yield Trial (ESWYT) and the p-values of the correlations (above the diagonals). a. The different shades of blue and red indicate different values of correlation presented in each component of the figure.

The average GY of Attila ranged from 2.0 t ha–1 in ME4 (33th ESWYT) to 6.31 t ha–1 in ME1 (31st ESWYT), whereas for LCs, the average GY ranged from 2.6 t ha–1 in ME4 (33rd ESWYT) to 6.76 t ha–1 in ME1 (29th ESWYT). Phenotypic correlations for GY were highly significant between ME1 and ME4 in the 28th, 29th, 31st, 33rd, and 34th ESWYTs (Fig. 2). Correlations between ME1 and M5 were highly significant in the 28th, 31st, 32nd, and 33rd ESWYTs (Fig. 2). Significant correlations between ME4 and ME5 were observed in the 28th and 33rd ESWYTs (Fig. 2).

### Grain Yield Gains across Locations in each ESWYT

**Fig. 3:**
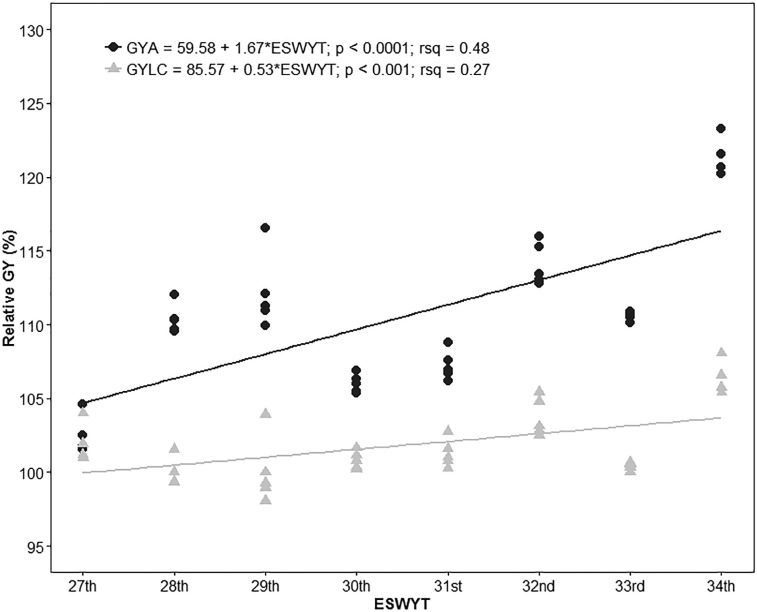
Relative wheat grain yield (GY) across locations of the 10% highest yielding lines in each Elite Spring Wheat Yield Trial (ESWYT) in relation to cv. Attila (GYA) and the local check (GYLC).

The HYL across the locations of each ESWYT showed that in the last 8 yr of international ESWYTs, there has been a significant GY increase of 90.1 kg ha^–1^1 yr^–1^1 compared with the cultivar Attila and 28.7 kg ha^–1^1 yr^–1^1 compared with the LCs, which represents an annual increase of 1.67 and 0.53%, respectively (Fig. 3). The estimates of the GY gains for GYA and GYLC without modeling the G × E interaction with the FA structure were 1.43 and 0.46%, respectively.

### Grain Yield Gains in ME1

**Fig. 4:**
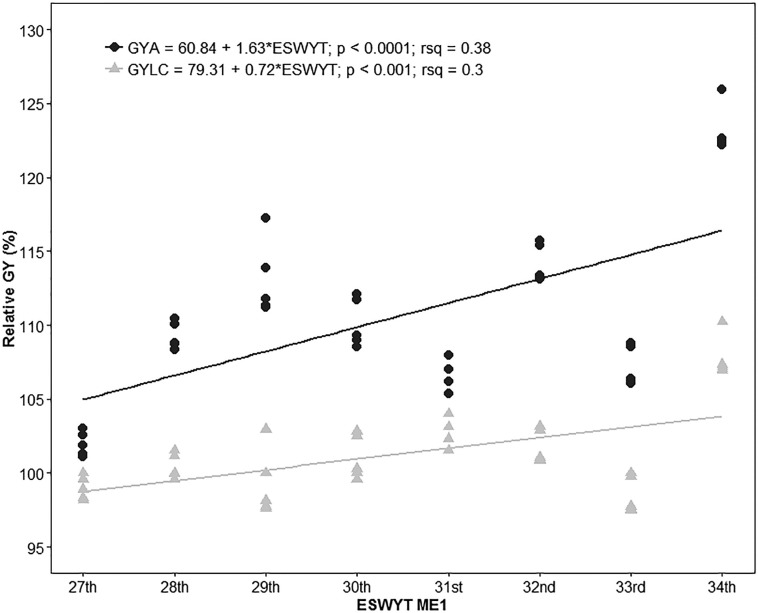
Relative wheat grain yield (GY) in Megaenvironment 1 (ME1) of the 10% highest yielding lines in each Elite Spring Wheat Yield Trial (ESWYT) in relation to cv. Attila (GYA) and the local check (GYLC).

Megaenvironment 1 had the highest number of locations, with yield data from 180 sites being classified as ME1 (Table 2), representing 59% of the total number of locations across all ESWYTS in all MEs. The analysis showed a significant increase in GY progress in ME1 (Fig. 4). The GYA showed an average increase of 1.63% or about 102.7 kg ha^–1^ yr^–1^ and 0.72% (46.65 kg ha^–1^ yr^–1^) for the GYLC. Estimates of the GY gains for GYA and GYLC without modeling the G × E interaction with the FA structure were 1.53 and 0.42%, respectively.

### Grain Yield Gains in ME4

**Fig. 5:**
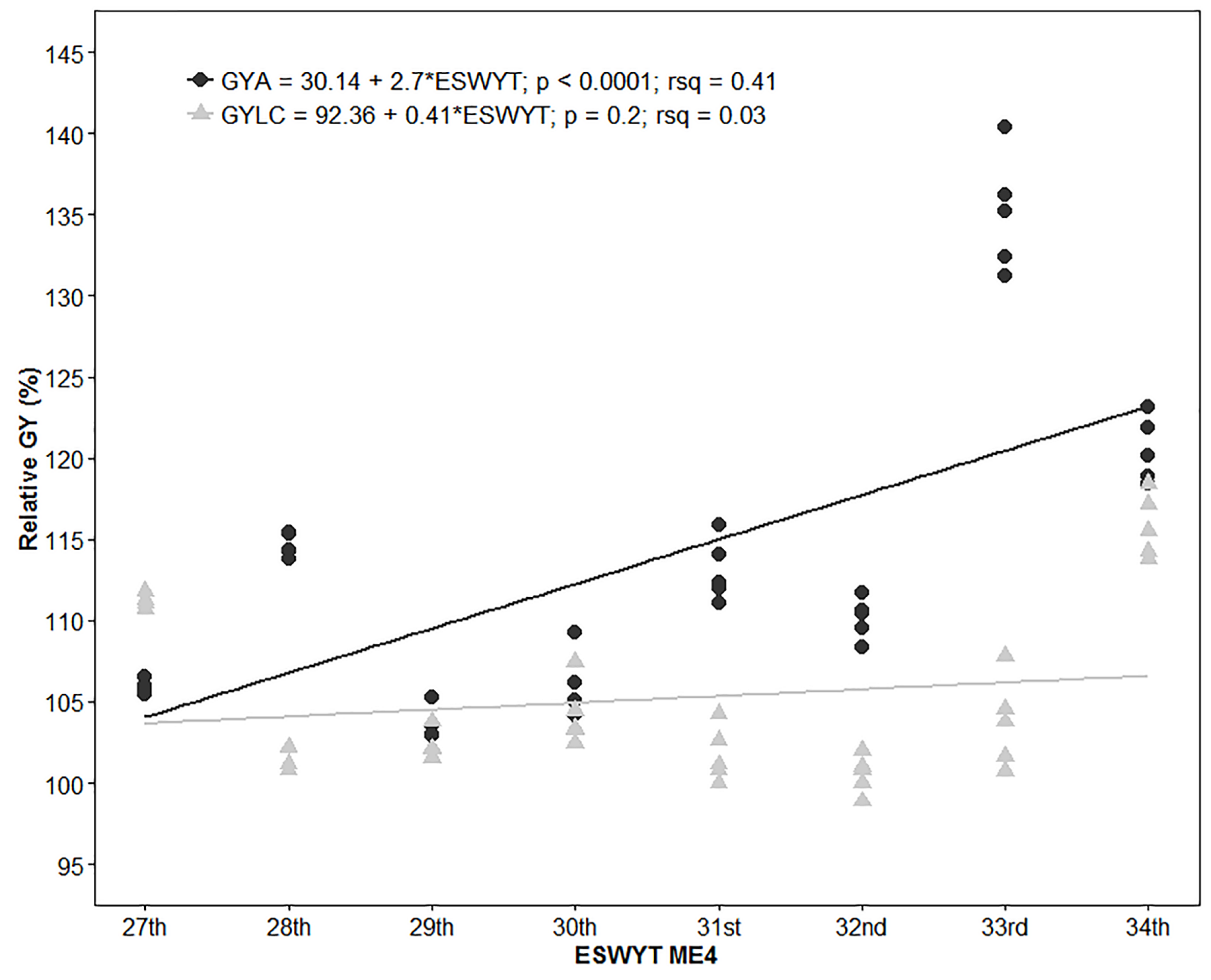
Relative wheat grain yield (GY) in Megaenvironment 4 (ME4) of the 10% highest yielding lines in each Elite Spring Wheat Yield Trial (ESWYT) in relation to cv. Attila (GYA) and the Local Check (GYLC).

Seventy locations represented ME4, which is 22.9% of the total analyzed (Table 2). The HYL in each ESWYT showed a significant annual GYA progress of 2.7% (Fig. 5), which, in terms of GY increase, is 88 kg ha^–1^ yr^–1^. The GYLC of the HYL had an increase of 0.41% (15.45 kg ha^–1^ yr^–1^). Estimates of the GY gains for GYA and GYLC without modeling the G × E interaction with the FA structure were 0.74 and 0.08%, respectively.

### Grain Yield Gains in ME5

**Fig. 6:**
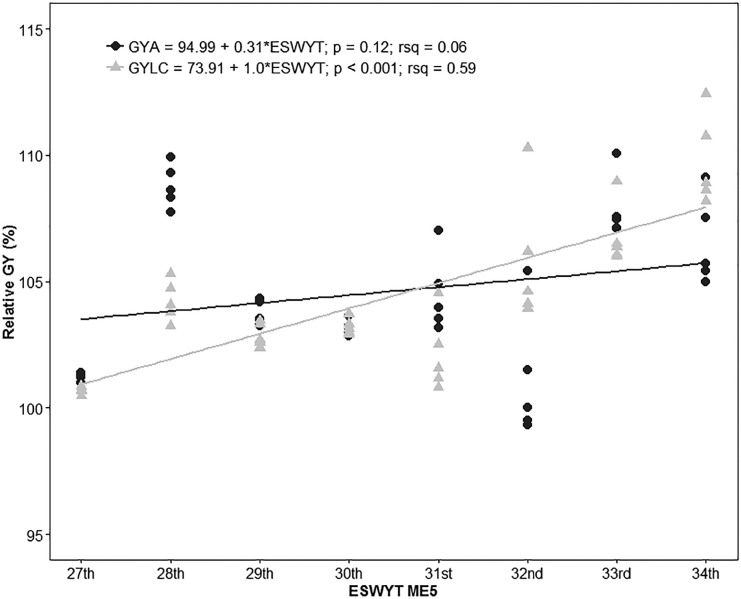
Relative wheat grain yield (GY) in Megaenvironment 5 (ME5) of the 10% highest yielding lines in each Elite Spring Wheat Yield Trial (ESWYT) in relation to cv. Attila (GYA) and the local check (GYLC).

There were 55 locations that represented ME5, which is 18% of the total (Table 2). Because the FA model did not fit the data in three of the ESWYTs (27th, 29th, and 32nd) and because BLUPS could not be estimated, we present the results of the analysis without using the FA structure. The trend of increase in terms of GYA was 0.31% or 11.28 kg ha^–1^ yr^–1^, whereas in terms of the GYLC, the observed significant rate of increase was 1.0% or 36.6 kg ha^–1^ yr^–1^ (Fig. 6).

### Grain Yield in the 34th ESWYT

The analysis of the 34th ESWYT showed that the HYL across MEs had between 20.2 and 23.3% higher GY than Attila, and between 5.4 and 8.1% higher yield than the LC (Table 3). The HYL in ME1 showed a GY advantage of 22.2 to 26% and 6.9 to 10.2% over Attila and the LCs, respectively. In ME4, the top five lines displayed a GY of 18.4 to 23.2% and 13.8 to 18.4% higher than Attila and the LCs, respectively. In ME5, the GY advantage of the HYL over Attila was 5.0 to 9.1% and 8.2 to 12.4% over the LCs. Pedigrees and ranks of the 50 genotypes included in the 34th ESWYT can be found in the Supplementary Material. Phenotypic correlations were statistically significant between ME1 and ME4 (*r* = 0.4; *p* = 0.01), whereas the correlation between ME1 and ME5 was not significant (Fig. 2). No correlation was observed between ME5 and ME4 (Fig. 2).

Some lines ranked as top performers in more than one ME. For example, line MUTUS*2/AKURI was the top performer in ME1 and ME5 and across locations. Line NAC/TH.AC//3*PVN/3/MIRLO/BUC/4/2*PASTOR/5/KACHU/6/KACHU was the top performer in ME4 and ME1 and across locations (Table 3). Line REEDLING #1, recently released in the Northwestern Mexico under the name “Borlaug 100 F2014”, was the top performer in ME1 and ME5 (Table 3).

**Table 3:** Grain yield (GY) in t ha^–1^, GY relative to the cultivar Attila (GYA), and GY relative to the local check (GYLC) of the highest yielding lines in the 34th Elite Spring Wheat Yield Trial (ESWYT) across all locations and by Megaenvironment 1 (ME1), ME4, and ME5.

Pedigree	GY	GYA	GYLC
Across MEs			
NAC/TH.AC//3*PVN/3/MIRLO/BUC/4/2*PASTOR/5/KACHU/6/KACHU	5.18	123.3	108.1
MUTUS*2/AKURI	5.11	121.6	106.6
K ACHU #1/4/CROC_1/AE.SQUARROSA (205)//BORL95/3/2*MILAN/5/KACHU	5.07	120.7	105.8
BECARD/QUAIU ^#^1	5.07	120.6	105.8
BECARD/FRNCLN	5.06	120.2	105.4
SE of the difference	0.08		
Grand mean	4.83		
LSD 5%	0.155		
CV %	1.64		
ME1			
K ACHU ^#^1/4/CROC_1/AE.SQUARROSA (205)//BORL95/3/2*MILAN/5/KACHU	6.18	126.0	110.2
N AC/TH.AC//3^*^PVN/3/MIRLO/BUC/4/2^*^PASTOR/5/KACHU/6/KACHU	6.02	122.7	107.3
REEDLING #1	6.01	122.5	107.2
KACHU//WBLL1*2/BRAMBLING	6.00	122.3	107.0
MUTUS*2/AKURI	6.00	122.2	106.9
SE of the difference	0.10		
Grand mean	5.66		
LSD 5%	0.20		
CV (%)	1.82		
ME4			
P BW343*2/KUKUNA/3/PASTOR//CHIL/PRL/4/GRACK	3.65	123.2	118.4
N AC/TH.AC//3*PVN/3/MIRLO/BUC/4/2*PASTOR/5/KACHU/6/KACHU	3.61	121.9	117.2
C HIBIA//PRLII/CM65531/3/MISR2, EGY/4/MUNAL #1	3.56	120.2	115.5
K AUZ*2/MNV//KAUZ/3/MILAN/4/BAV92/5/AKURI/6/MUTUS	3.52	118.8	114.3
BECARD/FRNCLN	3.51	118.4	113.8
SE of the difference	0.09		
Grand mean	3.23		
LSD 5%	0.19		
CV (%)	2.97		
ME5			
MUTUS*2/AKURI	5.05	109.1	112.4
SUP152/BAJ #1	4.98	107.5	110.8
SUPER 152	4.90	105.7	108.9
REEDLING #1	4.88	105.4	108.6
BECARD/QUAIU #1	4.86	105.0	108.2
SE of the difference	0.23		
Grand mean	4.67		
LSD 5%	0.44		
CV (%)	4.84		

The analysis of the locations assigned to ME1 in India, Pakistan, and Egypt showed a yield advantage of 43.4, 35.7, and 7.5% over Attila in these countries, respectively (Table 4). The highest GY gain over the LCs in these countries was 9.0, 17.5, and 11.9%, respectively. The number of locations was higher in India (11 locations), with only three in both Pakistan and Egypt.

**Table 4:** Wheat grain yield (GY) in t ha^–1^, GY relative to cultivar Attila (GYA), and GY relative to the local check (GYLC) of the highest yielding lines in India, Pakistan, and Egypt, classified as ME1 in the 34th ESWYT.

Pedigree	GY	GYA	GYLC
India			
MUTUS*2/AKURI	5.99	143.4	109.0
KACHU#1/4/CROC_1/AE.SQUARROSA (205)//BORL95/3/2*MILAN/5/KACHU	5.90	141.4	107.5
KAUZ*2/MNV//KAUZ/3/MILAN/4/BAV92/5/AKURI/6/MUTUS	5.80	138.9	105.6
BECARD/KACHU	5.71	136.8	104.0
SUP152*2/TECUE #1	5.61	134.3	102.1
SE of the difference	0.14		
Grand mean	5.30		
LSD 5%	0.27		
CV (%)	2.62		
Pakistan			
NAC/TH.AC//3*PVN/3/MIRLO/BUC/4/2*PASTOR/5/KACHU/6/KACHU	5.49	135.7	117.5
KACHU//WBLL1*2/BRAMBLING	5.33	131.7	114.1
SAUAL/3/ACHTAR*3//KANZ/KS85–8-4/4/SAUAL	5.29	130.8	113.2
KACHU#1/4/CROC_1/AE.SQUARROSA (205)//BORL95/3/2*MILAN/5/KACHU	5.27	130.4	112.9
ATTILA*2/PBW65*2//KACHU	5.24	129.5	112.2
SE of the difference	0.32		
Grand mean	4.52		
LSD 5%	0.63		
CV (%)	7.12		
Egypt			
MUTUS*2/TECUE #1	7.75	107.5	111.9
REEDLING #1	7.66	106.1	110.5
KACHU#1/4/CROC_1/AE.SQUARROSA (205)//BORL95/3/2*MILAN/5/KACHU	7.63	105.7	110.0
FRET2*2/BRAMBLING//BECARD/3/WBLL1*2/BRAMBLING	7.58	105.0	109.3
SUP152*2/TECUE #1	7.57	104.9	109.2
SE of the difference	0.38		
Grand mean	7.32		
LSD 5%	0.76		
CV (%)	5.25		

**Fig. 7:**
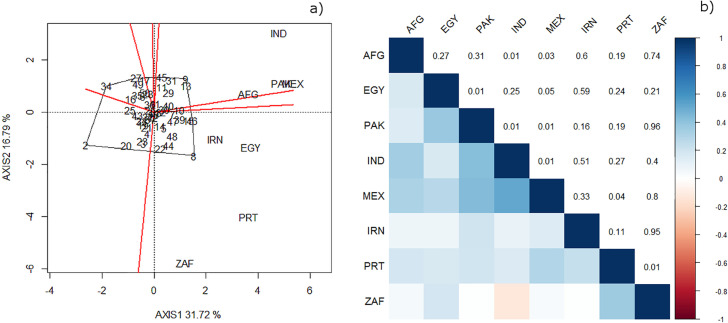
(a) Polygon in the biplot of the site regression analysis displaying the best performing wheat genotypes in the countries with locations classified as Megaenvironment 1 (ME1); (b) Pearson correlations (below the diagonal) among the countries in which locations were classified as ME1 and *p*-values of the correlations (above the diagonal). AFG, Afghanistan; EGY, Egypt; PAK, Pakistan; IND, India; MEX, Mexico; IRN, Iran; PRT, Portugal; ZAF, South Africa.

The first two components of the biplots derived from the site regression analysis model explained 48.51% of the total variation in ME1 (Fig. 7a). There were statistically significant correlation coefficients for locations in Mexico with Afghanistan, Egypt, Pakistan, India, and Portugal (Fig. 7a, 7b). There was no significant correlation for Mexico with either South Africa or Iran (Fig. 7b). The polygon drawn in the biplot with the most responsive genotypes as vertices indicated that India, Mexico, Afghanistan, and Pakistan can form a single group, in which the genotype with the lowest crossover interaction is KACHU #1/4/CROC_1/AE.SQUARROSA (205)//BORL95/3/2*MILAN/5/KACHU (No. 9 in the biplot, Fig. 7a), which showed a yield advantage over Attila of 41.4% and an advantage of 7.5% over the LCs in India (Table 4). Egypt, Iran, Portugal, and South Africa are in a separate group where the line REEDLING #1 (No. 8 in the biplot, Fig. 7a) was the best performer, with a GY advantage over Attila of 6.1% and an advantage of 10.5% over the LCs in Egypt. The cultivar Attila (No. 2 in the biplot) appeared in the farthest quadrant of the biplot with respect to the locations, indicating that the performance of this genotype was the lowest across the trials. Full pedigrees, ranks, and average GY in the eight countries can be found in the Supplementary Material.

## DISCUSSION

Our analyses show that there is a continuous increase in the grain yield of the wheat germplasm developed by CIMMYT’s Global Wheat Program. This finding is in line with previous studies on CIMMYT germplasm (Sayre et al., 1997; Lopes et al., 2012; Sharma et al., 2012). However, in our study we modeled the G × E interaction by fitting an FA structure to the variance–covariance **GE** matrix to estimate the BLUP of GY, which displayed higher estimations of the genetic gains in comparison with models that lacked the FA structure. Additionally, we classified the locations into MEs according to climate data and agronomic practices.

The cultivar Attila was used as a benchmark to evaluate GY progress because Attila is still grown on more than 5 million ha in Asia (Lantican et al., 2005; Singh et al., 2012), of which more than 2 million ha were planted in India in 2014 (Lantican et al., 2016); it also has yield stability and is suitable for growing in ME1, ME4, and ME5. However, new races of yellow rust fungus (caused by *Puccinia striiformis* f. sp. *tritici* Westend.), which are virulent to the resistance genes *Yr9* and *Yr27* (Singh et al., 2012; Tomar et al., 2014), affect this cultivar, making it less suitable as a reference in future evaluations of yield progress per se. Data on disease infections are not commonly returned to CIMMYT; however, in the 34th ESWYT, some cooperators did report yellow rust infections (between 60 and 100%) for Attila in India (six cooperators), Pakistan (four), and Afghanistan (two), whereas the only two yellow rust reports in the 27th ESWYT from India, indicated 0–10% of rust infection. The HYL averaged 5% of yellow rust infection in India, Pakistan, and Afghanistan in the 34th ESWYT.

The GY gains throughout the evaluation period can also be compared with the GY of the LCs. The LCs are important points of reference because they are presumably the best cultivars released in the areas where the trials are grown and continue to be changed over time, thus raising the standard for selecting high-performing lines (Singh et al., 2007). Moreover, in CIMMYT target countries, they are usually direct or indirect CIMMYT wheat descendant cultivars that have been released and commercialized on a regular basis (Lantican et al., 2016). Additionally, our analysis shows that the LCs generally performed better than Attila, particularly across environments and in ME1 and ME4. Yellow rust does not occur in ME5 because of higher temperatures.

When the locations were partitioned by MEs, the highest GY gain was observed in ME1, as expected. We observed a GY increase relative to Attila of 1.67% per annum across all locations and 1.63% in locations assigned to ME1. Other reports have shown that the rate of increase for commercial GY in wheat is less than 1% (Fischer et al., 2009, 2014; Fischer and Edmeades, 2010). In a different evaluation period (16th–30th ESWYTs), Sharma et al. (2012) found that for ME1 the progress in GYA was 0.55% (27.4 kg ha^–1^). These contrasting results can be attributed to the fact that the evaluation periods of the two studies were different and also to the use of the FA model for assessing the **GE** matrix; the FA model increases the precision of the predicted GY estimates in multienvironmental trials. Nevertheless, another important factor that influences the differences in the results between Sharma et al. (2012) and our analysis is the inclusion in the last four ESWYTs of newly developed elite lines that show a major advantage of GYA and GYLC over those in earlier international trials.

Although ESWYT germplasm is targeted at optimal environments, in ME4, we found 2.7 and 0.42% gains in GYA and GYLC, respectively. Another study of CIMMYT germplasm targeted at ME4, the Semi-Arid Wheat Yield Trial, reported that Attila was a high-yielding line in ME4 during the first years of Semi-Arid Wheat Yield Trial distribution (1994–2000). However, Attila was outperformed by germplasm developed later, which was often Attila-derived (Manès et al., 2012). The GY gains found by Manès et al. (2012) were approximately 1%; however, they used another wheat cultivar, Dharwar Dry, as a reference point. The fact that phenotypic correlations between ME1 and ME4 were positive and, in most cases, highly significant, shows, to some extent, that it is possible to breed simultaneously for irrigated and water-limited environments (Braun et al., 1996; Singh et al., 2007). The highest GYLC and lowest GYA gains of the HYL were observed in ME5, indicating that Attila performed better than the LCs in ME5 locations, which could also be caused by the absence of yellow rust.

Yield gains in wheat have been reported to be associated with an appropriate phenology of plants in the target environments, such as cooler canopy temperature, increased 1000-kernel weight, kernel number per m^2^ and harvest index (Sayre et al., 1997; Lopes et al., 2012; Mondal et al., 2013). However, more recent evidence suggests that genetic yield gains in CIMMYT germplasm are mainly a result of increased grain weight (Aisawi et al., 2015). Additionally, there are adaptive traits that are relevant for environments in which drought (ME4) and heat (ME5) are major constraints, such as earliness, early vigor, delayed leaf senescence, and wax for ME5 in particular (Cossani and Reynolds, 2012; Mondal et al., 2013) and water extraction from deep in the soil, accumulation of soluble stem carbohydrates, and increased water use efficiency for ME4 (Reynolds et al., 2005, 2007).

Furthermore, to raise yield potential, some authors (Reynolds et al., 2009, 2012) have suggested increasing radiation use efficiency, which will then improve photosynthetic capacity. Also, optimizing source–sink relationships in plants is necessary so that the assimilates are partitioned to increase spike fertility, grain number and size, and harvest index (Reynolds et al., 2009, 2012; Foulkes et al., 2011; Parry et al., 2011). All this can be achieved by applying strategic crossing schemes, high-throughput phenotyping, molecular marker-assisted breeding, and conventional breeding methods (Singh and Trethowan, 2007; Reynolds et al., 2009, 2012). Although several quantitative trait loci for yield and yield components have been mapped in the wheat genome (Kato et al., 2000; Quarrie et al., 2006; Snape et al., 2006; Mason et al., 2013), the genetic basis of yield potential is largely unknown. However, knowledge in this area is expected to increase once the full wheat genome sequence is available, along with a better understanding of the way genes interact to express complex traits such as yield (Reynolds et al., 2012).

Other authors have demonstrated that CIMMYT’s testing location at Ciudad Obregon, Mexico (27°37′N, 109°93′W), is highly correlated with other sites where wheat is grown (Braun et al., 1992; Trethowan et al., 2001, 2003; Lillemo et al., 2005). The locations in countries where the 34th ESWYT was distributed showed a high correlation with Mexico, except for South Africa. In line with this, we observed some genotypes that were top performers in more than one ME or country.

The approach followed by CIMMYT to breed for wide adaptation has had highly significant impacts on wheat production, so that it is estimated that 70% of cultivars grown in target countries are of CGIAR (CIMMYT or ICARDA) ancestry or direct CGIAR lines, creating an estimated economic benefit for CGIAR national partners’ wheat research of US$1.5 to 4.8 billion per year (Lantican et al., 2016).

CIMMYT’s international multilocation testing strategy allows periodic assessment of the yield progress achieved by breeding programs on a global scale. These evaluations of GY progress are a fundamental part of CIMMYT’s activities, since the global scenario requires breeders to set strategies to increase wheat yield potential as a high priority. More precise prediction of genetic values is achieved by modeling the G × E covariance structure by means of a parsimonious model (i.e., FA). These analytical methods, together with the high GY performance of newly developed wheat lines, demonstrate the success of CIMMYT’s wheat breeding program in increasing GY genetic gains over the last 8 yr. To speed up cultivar release and information-sharing with cooperators, Singh et al. (2007) proposed publishing the analyzed data collected from the multilocation testing derived from CIMMYT’s international network biannually, although the information is publicly available on CIMMYT’s website (www.cimmyt.org, accessed 26 Oct. 2016).

## CONCLUSIONS

From our study, we concluded that CIMMYT continues to deliver improved and widely-adapted germplasm to its target environments in developing countries. The average rate of GY increase across the locations in which the ESWYT was distributed from 2006–2007 to 2014–2015 was 1.67% relative to Attila, and 0.53% relative to the LCs. Because Attila has become susceptible to new yellow rust races, it has been replaced by new resistant and high yielding lines in subsequent ESWYTs. In this way, future yield progress can be measured with reference to a genotype without the influence of rust infections.

Global wheat production in 2014 was more than triple compared with that in 1961 in about the same harvested area (FAO, 2016), which is a clear indication of the continuous growth of wheat yields in farmers’ fields. In 2014, the global average wheat yield was 3.2 t ha^–1^, and about 2.5 t ha^–1^ in developing economies (FAO, 2016). However, there is urgent need to do more, as it is estimated that wheat yields need to grow at an annual rate of 2 to 3% in farmers’ fields to meet the expected demand in 2050 (Hawkesford et al., 2013). This yield increase is expected to be attained not only from breeding alone but also through a holistic approach in which cropping technologies and adequate policies come together to increase wheat yields in farmers’ fields sustainably.

CIMMYT’s international multilocation testing network is an unprecedented effort aimed at breeding wheat on a global scale (Trethowan and Crossa, 2007). However, cooperators could increase their contribution to multilocation testing by increasing the rate of data return, since the estimated data recovery is only 40 to 50%.

## Abbreviations:

BLUP,: best linear unbiased predictors;
ESWYT,: Elite Spring Wheat Yield Trials;
FA,: factor analytical;
G × E,: genotype × environment;
GY,: grain yield;
GYA,: grain yield relative to the cultivar Attila;
GYLC,: grain yield relative to local checks;
HYL,: the 10% highest-yielding lines in each Elite Spring Wheat Yield Trial;
ME,: megaenvironment.
